# Needs, benefits, and issues related to home adaptation: a user-centered case series applying a mixed-methods design

**DOI:** 10.1186/s12877-022-03204-2

**Published:** 2022-06-27

**Authors:** Chloé Schorderet, Catherine Ludwig, Frederic Wüest, Caroline H. G. Bastiaenen, Robert A. de Bie, Lara Allet

**Affiliations:** 1 School of Health Sciences, HES-SO Valais-Wallis, University of Applied Sciences and Arts Western Switzerland, 5 Chemin de l’Agasse, 1950 Sion, Switzerland; 2 Department of Epidemiology, Care and Public Health Research Institute CAPHRI, Maastricht University, Maastricht, Netherlands; 3 Geneva School of Health Sciences, HES-SO, University of Applied Sciences and Arts Western Switzerland, 47 Avenue de Champel, 1206 Geneva, Switzerland; 4 Geneva School of Landscape, Engineering and Architecture, HEPIA, HES-SO, University of Applied Sciences and Arts Western Switzerland, 4 Rue de la Prairie, 1202 Geneva, Switzerland; 5 Department of Community Medicine, University Hospitals and University of Geneva, Geneva, Switzerland

**Keywords:** Older adults, Aging in place, Home adaptation

## Abstract

**Introduction:**

Home adaptation can be a key contributor to successfully aging at home, allowing older adults to remain in a familiar environment while maintaining their quality of life and well-being despite progressing functional difficulties. Although several theoretical studies on home adaptations exist, the benefits of custom home adaptations remain poorly evaluated. The present study's primary aims were to explore older adults' expectations and needs regarding home adaptations and evaluate the impact of individualized home adaptations on quality of life, fear of falling, independence, and difficulties using adapted rooms. Its secondary aim was to describe the barriers and facilitators of home adaptation.

**Method:**

The 15 homes in this case series were adapted using an inclusive, interdisciplinary approach. Adaptations' effects were assessed using a parallel mixed-methods design. Quantitative and qualitative data were collected using questionnaires and semi-structured interviews. An architect and a health professional visited each home twice to assess the older adult's expectations and needs, evaluate the home's technical aspects, and co-create an adaptation plan with that study participant. They assessed the older adult's perceived quality of life, fear of falling, independence, and difficulties using the rooms needing adaptations. Inhabitants received two more visits after the adaptations (one or two months and six months later) to assess their benefits.

**Results:**

Most homes had their bathroom adapted. Participants reported improved safety, independence, ease of use, positive feelings, and comfort. They also reported lower perceived levels of difficulties during the activities of daily living in the adapted rooms (reductions of 93.4% [SD = 12.7] of bathrooms and 100% of kitchens), an improvement in quality of life of 9.8% (SD = 27.6), and a reduction in fear of falling of 12.5% (SD = 9.7).

**Conclusion:**

Home adaptations are beneficial to older adults' activities of daily living and improve their quality of life; however, several factors hinder the implementation of those adaptations.

**Supplementary Information:**

The online version contains supplementary material available at 10.1186/s12877-022-03204-2.

## Introduction

WHO projections suggest that by 2050, one in five people will be 60 years old or more [1]. Although life expectancies have been increasing for two centuries [2], this has been accompanied by the increased prevalence of chronic diseases [3–5] and frailty [6] among older adults. Indeed, there has been a concurrent decrease in the prevalence of perceived good or excellent health [7]. Functional limitations, such as difficulties bathing, showering, walking, or using public transport, are also commonly observed, although mainly in advanced old age (i.e., ≥ 80 years) [8]. Thus, aging can be characterized by a progressive loss of physical resources requiring the constant implementation of individual and collective measures to maintain optimal independence, well-being, and quality of life (QoL) [9]. Aging in place is recognized as a major contributor to these goals [10, 11]. Wiles et al. [12] reported that aging at home had many benefits for older adults, including maintaining their identity and habits.

However, for the person to be able to age at home, it is essential to consider their personal living environment. Lawton theorized that there was a mutual influence between a person's physical and social environment and their behavior, reporting that the weaker an individual's behavioral skills, the more their environment impacted them [13]. Older adults are, therefore, particularly affected by their environment. Environmental situations poorly adapted to their skills could lead to maladaptive behavior or individual failure as the individual is not able to face environmental challenges [14]. "Accessibility" is commonly used to define the set of factors that influence an individual's functioning within their environment [15]. It is also used to define whether their environment allows them to function independently, regardless of any physical or mental dysfunction [16]. Home adaptations are the measures implemented to increase a home's accessibility [17], improve older adults' activity patterns [18], and reduce the risk of injury from falls [19].

Home adaptation also meets certain political expectations because it represents a potential solution to the financial and social challenges of fulfilling the needs of a constantly growing older adult population. Although several studies have highlighted the benefits of home adaptations [20–22], to the best of our knowledge, none had evaluated older adults' expectations of the individualized adaptations that might allow them to age at home and then their perceptions of the effects of those adaptations. Given that most older adults want to age at home, adaptations could be a pertinent solution for those likely to experience functional difficulties, but the proven effects of adaptations on this population should be evaluated. We estimated that a user-centered approach was essential to ensuring that individualized adaptations responded to specific needs.

The present study thus explored the expectations and needs of home-dwelling older adults themselves, their family members, and the professionals involved in their home adaptation to evaluate the impact of home adaptations on older adults' QoL, fear of falling, independence, and difficulties using adapted rooms. A secondary aim was to describe any issues, facilitators, or barriers related to home adaptations for older adults.

## Methods

### Design

To achieve these aims, we decided to use a case series study and a parallel mixed-methods design. Quantitative data were collected via questionnaires and qualitative data were collected using semi-structured interviews. This occurred in parallel, but data were analyzed separately. Before starting home adaptations, qualitative data allowed us to better understand participants' needs and expectations and analyzing these helped to better understand and interpret the quantitative results.

The study protocol was submitted to Geneva's Cantonal Research Ethics Commission (CCER), which declared that the research project did not require its approval (response date: November 3, 2017).

### Participants

Inclusion criteria were being aged 65 years old or more, living at home in the Swiss cantons of Geneva, Vaud, or Valais, and being interested in adapting one's home to help improve one's daily life. Older adults who had suffered an accident in the month preceding the first contact with investigators were excluded so as to target usual aging only. Other exclusion criteria were the inability to understand questions and conduct a conversation in French or German during the interviews. Participants were recruited via housing corporations and home-care institutions that were contacted directly by the research teams as well as through calls for participation, and articles about the study published in various media in Switzerland's French-speaking regions.

### Data collection

Participants meeting the inclusion criteria were initially contacted using a telephone call, during which they were more fully informed about the study. On receiving their oral consent to participate, investigators scheduled a first visit to the potential participant's home. During this first visit, the older adult was fully informed about the study, orally and in written form. If they again agreed to participate, they signed the informed consent document. This first visit was conducted by two research group members—an architect and a health professional. The architect inspected each part of the older adult's home and assessed its accessibility using a checklist adapted from the guidelines in F Bohn's "Swiss architectural design standards for older adult habitats" [23], which contains questions about the neighborhood, accessibility, staircases, and each room's detailed characteristics. The health professional conducted the first interview (pre1) with the home-dwelling older adult; this interview's quantitative and qualitative assessments are described below. The research team then included the participants in the reflection process by discussing their proposed adaptations. These proposals were individualized, based on the participant's needs and expectations and on particular architectural constraints. The research team also had to ensure that potential adaptations were in line with the participants' financial situation since the adaptations were to be at the participants' own expense. In cases where the research team proposed several adaptations, priorities were chosen with the participant. The team was also open to proposals from participants interested in combining adaptations with home renovations. Once the research team and the participant felt they had met expectations and needs in an optimal way, the work was planned out. A second visit by the health professional was scheduled to reassess every variable and perform a second interview (pre2), just before the adaptations were carried out, to check whether the participant's situation had remained stable.

Once the adaptations were completed, a health professional conducted two additional health interviews (one between one and two months after the adaptations [post1], one six months later [post2]). These aimed to evaluate the adaptations' perceived impacts and changes using the quantitative and qualitative assessments described below. Healthcare professionals interviewed participants either face-to-face or by telephone, taking hand-written notes about their responses.

If the participant's home living situation required assistance from family, professional caregivers or housing staff, they were also contacted by the research team and integrated into the process if they so wished. If this was the case, they were informed about the study by telephone and asked for oral consent to participate. At a subsequently scheduled meeting, they were fully informed about the study, orally and in written form, and signed the consent document if they agreed to participate. Whenever possible, one interview with them was conducted before the adaptations and two afterward. All data were collected in French or German. Interviews were audio-recorded, and interviewers took notes. Interviewers checked their notes immediately after the interviews and, if necessary, listened to them again to complete them. Every care was taken to ensure that participants' quotes were reported precisely.

The questions in the qualitative and quantitative assessments described below related to the full week before the interview.

### Qualitative data assessment

- *Adaptation needs and expectations*: home-dwelling older adults were asked open-ended questions to identify their needs and discover whether they expected home adaptations to change their daily life, QoL, and independence or whether they expected negative changes and discomfort.
- *Adaptations' perceived impacts on daily life*, *QoL*, *and independence*: participants were asked open-ended questions to identify the impacts home adaptations had had on their daily life, QoL, and independence, or whether they had caused any discomfort.

The open-ended questions (see Appendix I) were developed by the research team based on existing literature and their experiences with home adaptations. The relevance and importance of the questions were then discussed with the experts in aging in place and home adaptation, who collaborated with the research team as external consultants*.*

### Quantitative data assessment

- Data for population description:

- *◦ Health status*: assessed using a four-point Likert scale ranging from "very bad" to "excellent".
- *◦ Health-related QoL*: measured using the sixth item in the three-level version (EQ-5D-3L) of the EQ-5D [24, 25]—a visual analog scale (VAS) ranging from "worst imaginable health state" (0) to "best imaginable health state" (100).
- *◦ Independence in the activities of daily living* (ADL): measured using the Katz independence scale [26], which contains items on bathing, dressing, toileting, transferring, continence, and feeding. For each item, 1 point means the person is independent, and zero means they require supervision, personal assistance, or total care. This scale is reported to have excellent test–retest reliability and excellent inter-rater reliability with older adults [27], and the literature reports its good predictive and construct validity [28, 29].

- Data for intervention assessment:

- *◦ Perceived difficulty in the ADL in the adapted rooms*: assessed using a VAS ranging from "no difficulty" (0) to "maximum difficulty" (10).
- *◦ Health-related QoL*: measured using the first five items of the three-level version (EQ-5D-3L) of the EQ-5D [24, 25] that assess the dimensions of "mobility", "self-care", "usual activities", "pain/discomfort", and "anxiety/depression" using a three-point Likert scale ranging from "no problems" to "extreme problems".
- *◦ General QoL*: assessed using a VAS ranging from "worst imaginable QoL" (0) to "best imaginable QoL" (100).
- *◦ Fear of falling*: assessed using the Falls Efficacy Scale–International (FES–I) questionnaire [30]. This self-reported questionnaire contains 16 items describing activities that the participant is asked to assess with a four-point Likert scale ranging from "not at all concerned" to "very concerned" by the fear of falling. Excellent test–retest reliability has been reported for this questionnaire for assessing community-dwelling older adults [31].
- *◦ Satisfaction with home adaptations*: assessed using a five-point Likert scale ranging from "very dissatisfied" to "very satisfied”.

In pre-adaptation interviews, family members or professionals involved in home care were asked which adaptations they considered essential and why, and what they thought about the adaptations the older adult participant wanted. After the adaptations, they were asked what effects the adaptations had had on the participant's daily life, whether they had had any negative effects, and whether they had changed anything in their involvement with the participant.

Data for the secondary aim (describing other issues, barriers, facilitators) was collected using various means. When an older adult refused or was reluctant to make adaptations during the first home visit, the research team asked them for reasons and took notes on their answer. Various data on issues, barriers, and facilitators to do with adaptations were collected during interviews with the older adults and their family, professional caregivers, or housing staff. Notes were kept on all the interactions with home-dwelling older adults, health professionals, and the administrative services with which we collaborated throughout the study.

### Data analysis

Descriptive statistics were used to describe participants' baseline characteristics (age, health status, health-related QoL, independence in the ADL) and post-adaptation changes to perceived difficulties in an adapted room, health-related QoL, general QoL, and fear of falling. Changes in each variable were calculated using the difference between pre-adaptation and post-adaptation scores divided by the pre-adaptation score. The pre-adaptation score was the mean of the two pre-adaptation interview scores (pre1 and pre2), as this situation remained relatively stable. The post-adaptation score was the mean of the two post-adaptation interview scores (post1 and post2), as this situation also remained relatively stable.

All qualitative data (expectations for adaptations, needs, perceived impacts of home adaptations) were analyzed using qualitative thematic analysis—a frequently used method of complementary analysis to quantitative methods that involves categorizing participants' statements into a number of themes related to the research’s principal and subsidiary themes [32]. A research team member identified the ideas emerging from each statement and classified them into the different dimensions of the main theme categories. The entire process was reviewed by a second research team member, who checked every category and dimension. The two researchers initially disagreed on naming three of the categories, but they came to a consensus after discussion and an exchange of opinions. This analysis aims to highlight the different ideas, reflections, and statements mentioned by participants. German interviews were translated into French for analysis, and our findings were translated into English.

The qualitative data collected from interviews with family, professional caregivers or housing staff were listed in a table, and a summary of their statements was made for each dimension.

Regarding data analysis related to our secondary aim (describing the issues, barriers, and facilitators of adaptations), each team member reviewed the entirety of the interview notes several times. Team members held several discussions to define the categorization of the issues, barriers, and facilitators of adaptations identified.

## Results

### Description of the population

Older adult participants' ages at the first interview ranged from 65 to 86, with a mean of 75.1 years old (SD = 7.3). Fourteen women and four men (18 people in 15 homes) were interviewed, with six cases involving tenants and nine involving owner-occupiers.

At baseline, participants perceived their health to be "fairly good" (*n* = 13), "excellent" (*n* = 1), "fairly bad" (*n* = 3), or "very bad" (*n* = 1). Most participants (*n *= 11) reported an overall health-related QoL (measured using the sixth item on a VAS ranging from "the worst health you can imagine" [0] to "the best health you can imagine" [100]) of at least 70, three reported a score above 60, three reported a score below 60, and one did not answer the question. Overall health-related QoL remained stable throughout the study (a mean difference of 0.19% between pre- and post-adaptation scores), implying that changes in perceived health were not influenced by the quantitative measures of the impacts of home adaptations. Before adaptations, although they had several perceived difficulties, all the participants had been independent when going to the toilet, but two participants had needed help washing, and one had needed help dressing.

### General results

Research team members visited 35 houses, of which 15 were adapted and assessed. The remaining 20 cases did not participate in the study for different reasons, such as financial problems, health problems, or lack of motivation. Table 1 presents the adaptations for each case included: one studio, 11 apartments, and three houses. The most common adaptations were installing a waterproof door in the bathtub for ease of access and transforming the bathtub into a shower. Others included installing ramps, leveling thresholds, transforming showers, improving lighting, electrifying or installing electric blinds, making electrical installations safer, installing a front door video intercom, renovating a kitchen, replacing a veranda window, installing a new toilet bowl with a water jet, and adapting access to the outside. In three cases, the bathroom was not just adapted but completely renovated, and in one case, a kitchen and dining room were completely modernized (Table 1: cases 7, 11, 14, and 15). In case 14, the bathroom was renovated and the kitchen storage spaces were reorganized. Work costs ranged from CHF 2,300 to CHF 35,000 for cases only involving adaptations and from CHF 13,000 to CHF 82,200 for cases where at least one room was completely renovated.

**Table 1:** Summary of cases included

No	HOME TYPE	OWNER STATUS	GENDER AND AGE	ADAPTATIONS	DURATION OF WORK	COSTS OF WORK
1	5-room apartment	Tenant	Woman: 73	-Installing waterproof door in bathtub -Improving lighting	1 day	3,120
2	2-room apartment	Tenant	Woman: 78	-Installing waterproof door in bathtub	1 day	3,000
3	1.5-room studio	Owner	Woman: 86	-Installing waterproof door in bathtub -Electrifying blinds -Making electrical installations safer -Installing a front door video intercom -Renovating the kitchen (replacing and adjusting height of fridge and microwave)	1 day	4,590
4	3-room house	Owner	Woman: 75	-Installing a shower instead of a bathtub -Replacing a veranda window and installing an electric blind	1 day	35,000
5	3-room apartment	Tenant	Woman: 86	-Installing waterproof door in bathtub -Installing a ramp at kitchen entrance	1 day	3,680
6	3-room apartment	Owners	Woman: 67 Man: 67	-Transforming bathtub into a shower -Installing ramps between the entrance and kitchen and between kitchen and dining room	1 day	10,000
7	3-room apartment	Owner	Woman: 76	-Renovating bathroom including installing bathtub with waterproof door and toilet bowl with a water jet	14 days	35,000
8	5-room apartment	Owner	Woman: 84	-Transforming bathtub into a shower	1 day	5,300
9	3-room apartment	Tenant	Woman: 86	-Renovating shower, levelling thresholds, and installing motion detection lamp	1 day	6,700
10	2.5-room apartment	Tenant	Woman: 65	-Installing waterproof door in bathtub	1 day	3,290
11	4-room house	Owners	Woman: 71 Man: 69	-Renovating kitchen and dining room, replacing 1^st^-floor window, and adapting access to the balcony (installing a patio door)	4 months*	82,200
12	5.5-room apartment	Owner	Woman: 73	-Transforming shower (lowering height of the bowl and installing a seat inside)	4 days	10,555
13	3.5-room apartment	Tenant	Man: 67	-Transforming seated bathtub into an Italian-style shower with a seat inside	3 days	2,300
14	5-room house	Owners	Woman: 76 Man: 83	-Renovating bathroom	6 months**	13,000
15	3.5-room apartment	Owner	Woman:70	-Renovating bathroom including transforming bathtub into a shower	3 months	25,000

^*^ Work delayed because of the Covid-19. ** Son's apartment renovated at same time

### Qualitative results

#### Expectations regarding adaptations

Participants' expectations regarding adaptations mentioned the dimensions of safety, ease of use, independence, comfort, and preventive measures. Categories with similar ideas were identified within these dimensions. Table 2 presents the dimensions and categories mentioned in participants' expectations.

**Table 2:** Dimensions, categories, and example quotes about expectations of adaptations

Dimensions	Categories	Example quotes
**Safety**	Feeling safe during activities	*"able to safely go out to water the geraniums."*
	Less fear of falling	*"less scared because I won't have to step over the bathtub edge anymore."*
	Indirect safety	*“less need to bend down to use and clean the oven."*
**Ease of use**	Place or activity-specific ease	*"able to get into the shower more easily."*
	General ease of use	*"make daily life easier."*
**Independence**	Maintaining independence	*"avoid the need for somebody's help to shower."*
	Ending dependency	*"no longer be dependent; no need to wait for someone to wash me anymore."*
	Independence for the dependent partner	*"I hope that my husband will be able to wash himself without my help."*
**Comfort**	Activity-specific comfort	*"I will feel better and less tired washing myself."*
	General comfort	*[We will have] "more space."*
	Inconvenience	*"noise and dust"*
**Preventive measures**	-	*"less worry about the future; we would like to stay in our home."*

Regarding safety, we identified the categories of *feeling safe during activities, less fear of falling*, and *indirect safety*. For the category of *feeling safe during activities*, one participant hoped he would be "*able to safely go out to water the geraniums,"* and another hoped to be "*more confident*" about showering after the adaptations. Regarding the category of *less fear of falling*, two participants hoped to experience less anxiety after the adaptations were made. The category of *indirect safety* referred to participants' statements not relating directly to safety but whose consequences might affect it. For example, one participant hoped to have "*more light*" and "*less need to bend down to use and clean the oven*" after the adaptations.

We identified the ease of use categories of *place or activity-specific ease* and *general ease of use*. The former refers to the ease of use related to a room, e.g., a bathroom, or an activity, e.g., showering or accessing a garden to water the flowers. Some participants expected to "*find it easier to use the bathroom*," to "*be able to get into the shower more easily*," or to be able to "*shower without problems*." The latter category refers to overall expectations about ease of use, e.g., participants hoped that adaptations would "*make daily life easier*."

Regarding independence, we identified the categories of *maintaining independence*, *ending dependency*, and *independence for the dependent partner*. In *maintaining independence*, several participants wanted to continue carrying out their activities without having to rely on external help. One participant, for example, indicated that she wanted to “*avoid the need for somebody’s help to shower*.” Regarding *ending dependency*, several participants looked forward to being able to wash themselves and no longer needing the help they had at the time of the interview. Regarding *independence for the dependent partner*, one participant hoped that her husband would be able to wash himself and no longer need her help after the adaptations.

In the domain of comfort, we identified the categories of *activity-specific comfort*, *general comfort*, and *inconvenience*. Under *activity-specific comfort*, participants expected to "*have a shower every day*," "*feel better and less tired*" when washing themselves, or "*be more comfortable in the shower*." In the *general comfort* category, participants expected to be "*more comfortable*," to feel "*less cold*" (in adapted rooms), or to have "*more space*" after adaptations were made. Regarding *inconvenience,* some participants noted the discomfort caused by adaptation work, such as "*noise and dust*," and one participant indicated that the work on their home was a "*stress factor*."

Regarding preventive measures, several participants indicated that the adaptations would be useful "*in the future*" and hoped to have "*less to worry about in the future*."

### Perceived impacts of adaptations

The same dimensions of the perceived impacts of adaptations were identified in the one-to-two month assessment and the one six months later, thus the two post-adaptation interviews' findings are presented together. The dimensions identified were safety, independence, ease of use, positive feelings, comfort, and anticipating the future. Table 3 presents the dimensions and categories related to the perceived impacts of adaptations.

**Table 3:** Dimensions, categories, and examples quotes about perceived impacts of adaptations

Dimensions	Categories	Example quotes
**Safety**	Less fear	*"I’m less apprehensive."; "I am less likely to fall"* [while showering]
	Feeling safe during activities	*"I can shower safely without being afraid."*
	Indirect safety	[I can] *"sit in the shower if needed."*
**Independence**	Regained independence	*"I can shower by myself, slowly." "Before, I couldn't do anything."*
	Dependent partner's renewed independence	*"He no longer needs my help to take a shower."*
**Ease of use**	Place or activity-specific ease	*"I no longer have to think about whether to take a shower or not; it's easy to shower myself now."*
	General ease of use	*"I find it easier to do everything."*
**Positive feelings**	Well-being	*"It puts me in a good mood." "Since I am better, everything is better."*
	Feeling motivated	*"I improved other things; it gave me other ideas."*
	Freedom	*"Being able to shower when I want changed my life."*
**Comfort**	General comfort	*"It is more comfortable to shower.”; "The whole thing is more pleasant."*
	Functionality	*"I have better access to my things in the kitchen cabinets."*
	Environmental quality	*"There’s more natural light in my kitchen.”; "I am convinced that I will have fewer drafts."*
	Practicality	*"The toilet bowl's little jet* […] *it's super practical."*
	Inconvenience	*"noise and dust"*
**Anticipating the future**	-	*"I'm glad to know that it's all done and everything's in order, and that I can stay at home even if my health declines."*

We identified the safety categories of *less fear, feeling safe during activities,* and *indirect safety*. Regarding *less fear*, participants mainly reported being less fearful about falling and carrying out activities like stepping into the shower or going out on the balcony. For *feeling safe during activities*, participants indicated that they felt particularly safer when showering; one reported, "*I can shower safely without being afraid to fall over.*" The *indirect safety* category included statements not directly related to safety but influencing it, e.g., a participant who could "*sit in the shower if needed.*" Another participant reported that she previously had to step out of a window by climbing on a chair to access a kind of balcony where she had a lot of flowers. Taking care of this colorful environment was very important to her, and she reported that "*everything is easier*" since the adaptation.

We identified the categories of *regained independence* and the *dependent partner's renewed independence*. Regarding the former, several participants reported that since adaptations, they could shower without help. Regarding the latter, one participant who previously had to help her husband shower reported that since adaptations, he could do it independently, which was a considerable change for her.

We again identified the categories of *place or activity-specific ease of use* and *general ease of use*. Regarding the former, participants reported that it was easier to get in and out of the bathtub, wash themselves, and go out onto the balcony. One participant noted, "*I no longer have to think about whether to take a shower or not; it's easy to shower myself now*." Regarding the latter category, several participants reported that a range of things was easier since adaptations had been made, with one reporting "*I find it easier to do everything*" and another saying "*things are easier now*."

In the domain of positive feelings, we identified the categories of *well-being*, *feeling motivated*, and *freedom*. Under *well-being*, several participants reported feeling better overall since the adaptations. For example, one participant indicated feeling “*better about herself,*" and another reported, "*Since I am better, everything is better.*" Regarding *feeling motivated*, several participants reported that the adaptations motivated them to undertake other activities: "*I thought about doing other things: changing curtains, putting up blinds and screens. I still have plans.*" Regarding *freedom*, several participants reported that they appreciated showering when they wanted to.

Regarding the dimension of comfort, we identified the categories of *general comfort, functionality, environmental quality, practicality, and inconvenience*. Under *general comfort*, several participants indicated that they appreciated the new comfort experienced in their adapted rooms, especially in kitchens and bathrooms, and that it was "*more comfortable to shower*." Regarding *functionality*, several participants whose kitchens were adapted indicated that they had better access to their utensils and storage spaces. Regarding *environmental qualities*, several participants reported more light and more pleasant room temperatures following their adaptations. Under *practicality*, several participants reported the practical aspects of their adaptations, e.g., one participant said that the removal of thresholds was "*practical*," and another said that the water jet inside their toilet bowl was "*very practical*." Under *inconveniences,* several participants reported noise and dust while their work was being done, and one participant was unhappy with the ramp installed at her kitchen entrance, finding it "*too steep*" and having it removed.

Under anticipation, one participant reported, e.g., that she was happy to know that everything was "*done, and everything's in order*" and that she could stay in her home even if her health were to decline. Another participant said that the adaptations enabled her to think about which adaptations might be made in the future.

### Quantitative results

#### Changes in the perceived difficulty of the ADL in adapted rooms

The perceived level of difficulty of carrying out the ADL before and after bathroom adaptations, as measured using a VAS, fell by 93.4% (SD = 12.7). Every participant whose kitchen had been adapted no longer had any difficulties in it at all. The couple whose access to the outside was adapted noted that their difficulties going outside had disappeared completely.

#### Changes in health-related QoL

As measured using the EQ-5D-3L's first five items, adaptations reduced participants' difficulties across the different dimensions of health-related QoL. Comparing the first pre-adaptation (pre1) and the last assessment (post2), several people moved from level 2 (some problems) or level 3 (extreme problems) to level 1 (no problems) for the dimensions of mobility, usual activities, and self-care (see Table 4). There was great variability between the different assessments for the dimension of pain/discomfort and an overall increase in perceived pain/discomfort over time. For the dimension of anxiety/depression, findings were relatively stable throughout the study.

**Table 4:** Health-related QoL on the different dimensions of the EQ-5D-3L

	**Mobility**				**Self-care**				**Usual activities**				**Pain/discomfort**				**Anxiety/depression**			
Interview	A	B	C	D	A	B	C	D	A	B	C	D	A	B	C	D	A	B	C	D
Level 1: no problems / no pain or discomfort / not anxious or depressed	7	6	7	8	15	12	15	16	9	7	12	11	4	6	5	4	13	10	13	13
Level 2: some problems / moderate pain or discomfort / moderately anxious or depressed	9	7	7	8	2	2	0	0	7	7	4	5	9	5	8	5	4	4	5	4
Level 3: many problems / extreme pain or discomfort / extremely anxious or depressed	2	2	3	1	1	1	2	1	2	1	2	1	5	4	5	8	1	0	0	0

Numbers correspond to the number of people reporting a perceived level of difficulty during each interview. A = pre1, B = pre2, C = post1, D = post2

### Changes in overall QoL

Overall QoL was assessed using a VAS ranging from 0 to 100. A mean improvement of 9.8% (SD = 27.6) was observed before and after the adaptations.

### Changes in fear of falling

Regarding fear of falling, an improvement of 12.5% (SD = 9.7) was measured using the FES–1 before and after the adaptations.

### Satisfaction with adaptations

**Fig. 1:**
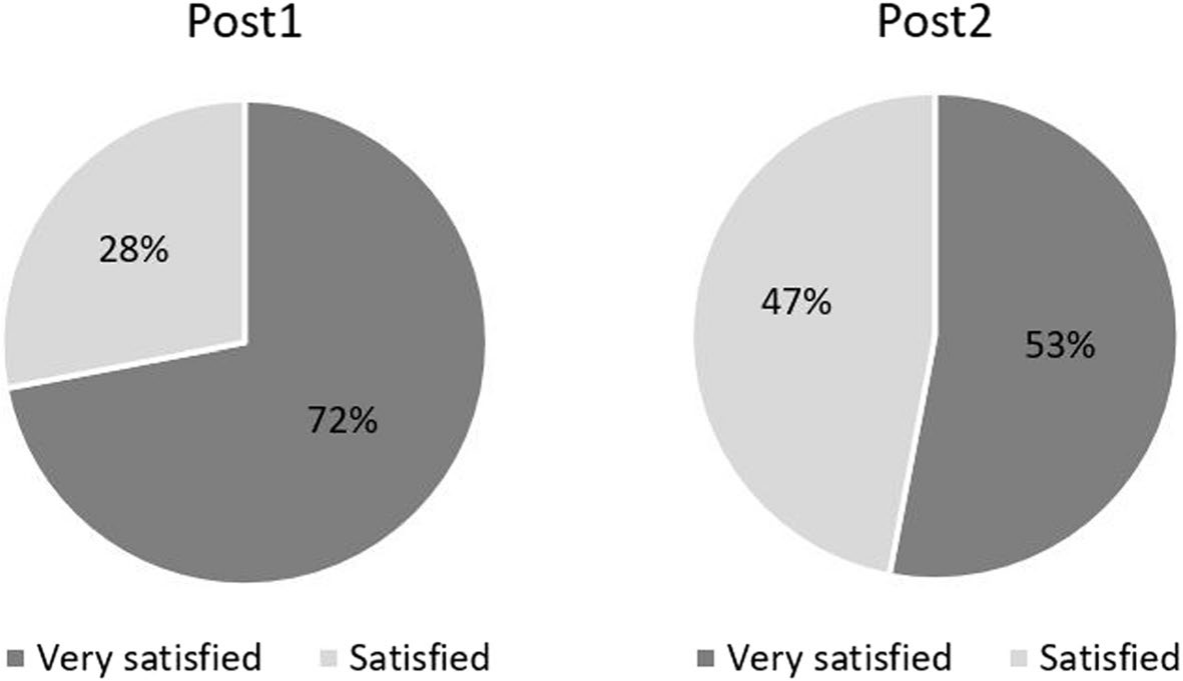
Satisfaction with adaptations. Percentage of people satisfied and very satisfied with the adaptations at the first and at the second assessment after the adaptations

Figure 1 presents results on satisfaction with adaptations, assessed using a Likert scale ranging from "very dissatisfied" to "very satisfied," with 72% of all participants reporting being very satisfied and 28% being satisfied at the first post-adaptation assessment. During their second post-adaption assessment, 53% of participants reported being very satisfied and 47% being satisfied.

### Results of interviews with family, professional caregivers and housing staff

*Family, professional caregivers and housing staff* were interviewed in four cases, and their answers were consistent with those of their corresponding home-dwelling older adult participants. They identified notions of independence, safety, and comfort.

*Case 1*: Prior to the adaptations, the cleaner reported that bathroom renovation would be "*practical*" for the participant, and she believed that installing a front door video intercom would be "*good,*" increasing the older adult’s safety. She added that she had a good feeling about the study.

*Case 2:* The cleaner reported that she did not see any "*obstacles*" in the participants' home now but that she found it good that the participant was thinking about the future as the use of the shower might become difficult when getting older. She added that converting the bathtub was a good idea. After the adaptations, she reported that the participant was very happy, that it was "*easier for her,*” and that it was “*good that she made the decision to make adaptations.*".

*Case 3*: Before the adaptations, the older adult participant's daughter, cleaner, and occupational therapist reported that she could no longer get in or out of the bathtub alone and that they thought installing a ramp would be good for safety. The cleaner remarked that the older adult's main risk was falling; the occupational therapist said that adaptations were needed to reduce this risk. The daughter thought that "*leaving a person at home is more beneficial than putting them in a specialized institution.*" Post-adaptation, regarding the bath, the occupational therapist reported that she added a bar to the wall and put anti-slip strips in the bath. The daughter reported that her mother was now more independent, able to get into the bath independently, and no longer needed her help to shower. The daughter and the occupational therapist reported that the newly installed kitchen entrance ramp was too steep, unfortunately, and that the older adult did not use it because she did not feel safe.

*Case 4*: Before the adaptations, the cleaner reported that the older adult's main risk was falling (particularly due to carpets and clutter in the flat). She thought that leveling the thresholds and installing a ramp were "*good ideas*" and that adapting the shower would be "*useful.*"

### Observations regarding issues, facilitators, and barriers to home adaptations for older adults

During the study's different phases, the research team's interactions with older adult participants, property owners, health professionals, and administrative bodies revealed many observations regarding issues, facilitators, and barriers to home adaptations. Table 5 presents the barriers to home adaptations.

**Table 5:** Categories of identified barriers to home adaptations

**Barriers related to the social-health system**	
*Administrative factors*	The numerous administrative procedures related to home adaptations were barriers to older adults interested in adapting their homes
*Financial costs*	Older adults frequently identified the cost of the work as a barrier, especially if they had limited resources. The fact that home adaptation costs are the older adult's responsibility and are not reimbursed by any type of insurance is a major obstacle to their implementation
*Access to information*	Older adults found it difficult to know where to get clear information about the home adaptation options available. The fact that there was no specific body to inform them about home adaptation options and procedures contributed to this problem
**Barriers related to professionals**	
*Lack of interprofessional collaboration*	The research team identified a lack of collaboration between health professionals and building professionals
*Insufficient number of building professionals doing home adaptations*	The research team found that few building professionals were trained in home adaptations for older adults or had experience in this area
**Barriers related to housing authorities**	
*Requirement to restore a home in its original state*	Most property owners in Switzerland require that their property be restored to its original state when tenants leave, creating a significant barrier for many older adults interested in adapting their home

The research team also observed that family members played a key role in motivating older adults to adapt their home, but they could be both barriers and facilitators. Some family members supported the older adult in their choice, encouraging them to undertake home adaptations quickly; other family members were reluctant to make adaptations, which tended to discourage the older adult and make them doubt their need for them.

Finally, one major issue was the difficulty of defining the right time to undertake home adaptations. The research team found that older adults' difficulties varied depending on their age and level of dependence. Those who were recently retired and in good health felt that it was too early to undertake adaptations, whereas those who were older and less independent felt that they no longer had the energy to start work on their home.

## Discussion

The present study's main aims were to identify older adults' expectations and needs regarding home adaptations and evaluate the perceived impacts and changes resulting from individualized home adaptations. Qualitative findings revealed that older adults had expectations and needs regarding safety, ease of use, independence, comfort, and preventive measures, and the home adaptations executed met them. Indeed, our qualitative findings on the adaptations' impacts highlighted the same dimensions. We identified benefits regarding safety, independence, ease of use, positive feelings, comfort, and anticipating the future. Quantitative results highlighted the considerable reduction in older adults’ perceived levels of difficulties during the ADL in rooms that had been adapted (93.4% reduction in difficulties for those who adapted their bathroom and a 100% reduction for those who adapted their kitchen and outside access), improvements in health-related QoL and general QoL, and a reduction in fear of falling.

The study's secondary aim was to describe issues, barriers, and facilitators related to home adaptation. The research team identified barriers related to the social-health system, professional limitations, housing authorities, and older adults themselves. Another key issue is identifying the right time for home adaptations: not too early, as nobody likes to admit that they are getting older, but not too late, so as to still have the energy to start a home adaptation project. Many participants said that carrying out adaptations was a great idea, but they did not yet feel concerned as they were still in good shape. Yet we also observed that as soon as the first functional problems limited an older adult's daily life activities, it was difficult for them to find the energy to think about a home adaptation project—they often declared that making adaptations was probably no longer worthwhile. This is regrettable, as the older adults with the most functional difficulties in their daily lives would surely reap the most benefit from adaptations. Thus, older adults themselves can be a barrier to adaptations. It would seem beneficial to develop a strategy for informing older adults about home adaptations as early as possible, raising awareness of the need for making such adaptations early on, while they still have the energy to initiate the process. It might be advantageous for home adaptation professionals to think about attractive design solutions as improved esthetics might encourage older adults to carry out adaptations for preventive purposes.

Another issue seemed to be the necessity to compromise between ideal adaptations and an affordable solution: financial constraints were often an obstacle. Indeed, after the first visit by team members, three older adults decided not to participate for this reason, and in several cases, not all the desired adaptations could be carried out—priorities had to be made to keep within budget. We also found that older adults who owned their homes did not approach performing adaptations in the same way as tenants. Indeed, tenants had more constraints, and their choices were sometimes limited, notably because of the conditions imposed by the property owner or housing authority. Older adult homeowners had more flexibility and sometimes combined adaptations with entire room renovations. Finally, family members were identified as either barriers or facilitators of home adaptations, depending on their perspectives.

Using a mixed-methods design enabled us to respond specifically to participants' needs and expectations and obtain comprehensive results. Qualitative data from the first pre-adaptation assessments enabled us to understand older adult participants' concrete needs and expectations regarding adaptations for their homes. This allowed the research team to propose targeted interventions to meet individual needs. The discussions about participants' expectations and needs made them feel like fully integrated partners in the process. Analysis of our qualitative findings contributed to a better understanding of the nature and reasons for our quantitative results, such as the lower perceived levels of difficulty in the ADL in adapted rooms, especially bathrooms. Qualitative results helped us identify that improvements in perceived QoL and general QoL were due to different benefits resulting from the adaptation, such as improved safety, ease of use in daily life, and independence. Indeed, older adult participants very often mentioned that being independent was an essential component of having a good QoL, and as most of the adaptations had this goal, it seems likely that this was reflected in our quantitative measures of QoL. We also believe that the positive feelings and comfort resulting from the adaptations were the sources of a motivational boost that encouraged several older adults to become more active. This increased energy certainly also played a key role in the improvements to QoL. The lower fear of falling revealed by the FES–I can be explained by the fact that participants felt safer taking a shower or moving around their apartment.

Although several older adults said that it was too early for them to envisage home adaptations, a relatively high proportion made them in anticipation of their potential future expectations and needs. This was probably because most of the older adults in the study were recruited through calls for participation and articles about the study published in various French-language media in Switzerland. Most of the participants indicated that they had a “*fairly good*” QoL during their first interview, and very few were dependent on external help for the ADL. This may help explain why we were only able to include family, professional caregivers or housing staff in four cases. It also suggests that people want to age at home and thus anticipate their future needs. Indeed, inhabitants sought to ensure aging in place as autonomously as possible because they saw living independently at home as synonymous with good QoL. The situation may well have been different if most of the older adults had been recruited via a home care institution, for example.

Our results aligned with previous literature reporting the benefits of home adaptations and fewer difficulties in daily activities [22, 33]. Benefits in terms of independence, QoL, and fall efficacy were also identified [21, 33]. Finally, they also reported that home adaptations for people with health disorders had positive effects on safety, autonomy, function, and independence [34, 35].

Some of our results were also in line with papers concerning issues, barriers, and facilitators related to home adaptations. Like us, different authors have highlighted that financial aspects contributed significantly to decisions to undertake home adaptations [36, 37]. The issue of the difficulty of choosing the right time to undertake home adaptations has also previously been discussed in the literature. Different authors have reported that the older people became, the more likely they were to make home modifications or relocate [38, 39]. A study by Thordardottir et al. [20], investigating the participation in everyday life of people who had benefited from housing adaptations, highlighted an additional issue: being independent in daily life at home will require modifications due to changes in health status and needs over time [20]. To remedy this difficulty, particular attention should be paid to assessing home-dwellers' level of participation in everyday life before undertaking home adaptations [20]. However, none of these authors reported any kind of a missed timepoint or situation of no return when people suddenly feel too old to adapt their home.

To the best of our knowledge, this study was the first in which health professionals and architectural professionals collaborated closely to evaluate and then implement home adaptations to meet older adults' individualized needs with the aim of enabling them to remain in their homes for as long as possible. This interprofessional collaboration, combining the domains of health and building, was an important strength of the study. Another was its inclusive approach focusing on older adults' individual, specific needs to make custom home modifications. A further strength was the collection and analysis of qualitative and quantitative data from two pre-adaptation and two post-adaptation interviews, enabling us to obtain very comprehensive results on many aspects of the changes resulting from home adaptations. The inclusion of 15 cases, each different in terms of place of residence (urban, peri-urban, rural), type of home (studio, apartment, house), financial relationship to the property (homeowner, tenant), type of adaptation (minor, major, renovation), and family situation (single, couple), was also a strength. It provided a broad overview of the situation in French-speaking Switzerland. Finally, the fact that we assessed the whole home unit and not just one particular room also represents a strength.

We are, nevertheless, aware that our study had some limitations. Our case-series methodology had two major limitations influencing qualitative data collection. Firstly, only one team member identified the categories and dimensions. Although a second member checked all the analyses and they searched for a consensus in cases of disagreement, the preferable option is to include a third person; however, this was impossible in this situation. Secondly, interviews were not transcribed. Analysis of the qualitative data was based on the interviewer's notes. Interviews were audio-recorded, however, and the researcher reviewed her notes and completed them by listening to the interviews again if necessary. In a larger study with fewer methodological limitations, verbatim transcripts would be the first choice. To limit bias as much as possible, particular care was taken to ensure that participants' quotes were accurately reported. Another limitation was the relatively homogeneous study population. Older adult participants were relatively healthy and independent. However, our aim was to explore the expectations and impacts of housing adaptations in French-speaking Switzerland, independently of the participants' functional abilities. It would be interesting to conduct another study on this topic, with a less independent population that would probably benefit more from such home adaptations. A further significant limitation was that the study participants had to pay for the adaptations themselves, which certainly excluded many older adults. Results should only be interpreted with regards to similar populations.

The potential benefits of home adaptations for older adults should be emphasized widely, and bodies to inform them about how these could boost their independence and chances of aging at home should be developed. Adaptations should be promoted to homeowners, especially by making them realize that an adapted home could be a valuable selling point in the search for new tenants, especially older adults. The research team observed that homeowners were often reticent about adapting their homes, however. In addition to the many benefits for current older adult tenants, adapted homes could also be attractive to future tenants, especially since many adaptations could be made at a relatively low cost.

As worldwide populations age, we believe that establishing interdisciplinary training programs for architects and health professionals will become essential. Skills in both domains are necessary to provide quality, comprehensive services to people wishing to adapt their homes. In this context, setting up show apartments to display the many different possible adaptations would not only be relevant for teaching purposes but also to show older adults looking for concrete examples of the latest beneficial adaptations.

The research team also considers that political reflection on financing options is essential. It is regrettable that costs seem to be a barrier when home adaptations can save on so many other care-related costs. Allowing older adults to age in place independently could prevent or reduce the need for home care services and delay or avoid institutionalization. In addition, home adaptations could reduce very costly hospitalizations due to falls. Hwang et al. [11] reported that an older adult whose house was adapted was more likely to remain at home, saving on the significant costs of hospitalization. The study by Mann et al. [40] supports this: they reported that using assistive technology and environmental interventions slowed functional decline, leading to reduced healthcare costs [40]. The most common adaptation carried out in our study was the installation of a watertight door in the bathtub, costing about CHF 3,200 on average. This corresponds to the average cost of a ten-day stay in a nursing home in Switzerland [41] or one hour per day's home-care help with hygiene (bathing, getting up, going to bed) [42].

## Conclusion

The present study described older adults' expectations of home adaptations and perceptions of their benefits regarding safety, independence, ease of use, positive feelings, comfort, and anticipating the future. Participating older adults also reported reductions in perceived levels of difficulty during the activities of daily living in the rooms they had adapted, improved health-related quality of life and general quality of life, and reduced fear of falling. However, although the benefits of adaptations are real and represent a cost-effective alternative to institutional care, carrying them out remains challenging, notably because of various human obstacles related to older adults themselves, health professionals, and homeowners. We therefore recommend collaboration between the public, private, and not-for-profit sectors concerned to set up specific bodies to help older adults remain in their homes, age in place, and gain in independence and quality of life. We also encourage the various health professionals working in the community and home care to collaborate to fully assess the environmental factors affecting older adults and to consider them in their treatment plans.

## Supplementary Information

**Additional file 1. Appendix 1** 

**Additional file 2. Appendix 2**

**Additional file 3. Appendix 3**

**Additional file 4. Appendix 4** 

## Data Availability

The datasets used and analyzed during the present study are available from the corresponding author on reasonable request.

## Abbreviations

ADL: Activities of daily living
CCER: Cantonal Research Ethics Commission
FES–I: Falls Efficacy Scale–International
IADLs: Instrumental Activities of Daily Living
VAS: Visual analog scale
