# Supplementary material for: Needs, benefits, and issues related to home adaptation: a user-centered case series applying a mixed-methods design
Source: BMC Geriatr. 2022 Jun 27;22:526. doi: 10.1186/s12877-022-03204-2 (PMC9235135; doi:10.1186/s12877-022-03204-2)
Supplement: Supplementary file 1 — Additional file 1. Appendix 1 [file 12877_2022_3204_MOESM1_ESM.pdf]

APPENDIX 1

Qualitative data assesment

Needs and expectations for adaptations:

- Do you think that the adaptations that will be made to your home will lead to changes in your daily life?
- Do you expect changes in terms of quality of life?
- Do you expect changes in terms of independence?
- Do you think that the adaptations that will be made may create inconvenience or have negative effects for you?

Perceived impacts of adaptation on daily life, independence, and quality of life:

- Do you think that the adaptations made in your home have led to changes in your daily life?
  If yes, please specify which ones and give concrete examples.
- Do you think that the adaptations made to your home have led to changes in your quality of life?
  If yes, please specify which ones and give concrete examples.
- Do you think that the adaptations made in your home have led to changes in your independence?
  If yes, please specify which ones and give concrete examples.
- Do you think that the adaptations made in your home have led to other changes than to your daily life, quality of life or independence?
  If yes, please specify which ones and give concrete examples.
- Did the adaptations made cause any inconvenience or have any negative effects for you? If yes, please specify which ones and give concrete examples
