# Supplementary material for: Needs, benefits, and issues related to home adaptation: a user-centered case series applying a mixed-methods design
Source: BMC Geriatr. 2022 Jun 27;22:526. doi: 10.1186/s12877-022-03204-2 (PMC9235135; doi:10.1186/s12877-022-03204-2)
Supplement: Supplementary file 2 — Additional file 2. Appendix 2 [file 12877_2022_3204_MOESM2_ESM.pdf]

APPENDIX 2

Perceived difficulty during ADL in the adapted rooms

N° part.	Pre1	Post1	Post2	Reduction in perceived difficulty (%)
**BATHROOM**				
1	10	-	0	**100**
2	5	0	0	**100**
3	10	0	0	**100**
4	0	0	0	**-**
5	10	0	0	**100**
6	-	0	0	**-**
7	0	0	0	**-**
8	6	0	0	**100**
9	3	0	0	**100**
10	5	0	0	**100**
11	10	0	-	**100**
12	4.5	3.5	0	**61**
15	9	1.5	0	**92**
16	-	7	0	**-**
17	8	0	4	**75**
18	7	0	-	**100**
**Mean improvement (%)**				**93.4**
**KITCHEN**				
3	8	0	-	**100**
12	8	0	0	**100**
13	8	0	0	**100**
**Mean improvement (%)**				**100**
**ACCESS TO OUTSIDE**				
12	8	0	0	**100**
13	8	0	0	**100**
**Mean improvement (%)**				**100**
