# Supplementary material for: Needs, benefits, and issues related to home adaptation: a user-centered case series applying a mixed-methods design
Source: BMC Geriatr. 2022 Jun 27;22:526. doi: 10.1186/s12877-022-03204-2 (PMC9235135; doi:10.1186/s12877-022-03204-2)
Supplement: Supplementary file 4 — Additional file 4. Appendix 4 [file 12877_2022_3204_MOESM4_ESM.pdf]

APPENDIX 4

Fear of falling

Quality of live (VAS)					
**N° Part.**	Pre1	Pre2	Post1	Post2	**Improvement (%)**
1	-	60	70	80	**25**
2	70	60	40	60	**-23.1**
3	80	75	88	95	**18.1**
4	90	90	95	90	**2.8**
5	50	80	40	50	**-30.8**
6	80	80	90	90	**12.5**
7	80	99	95	90	**3.4**
8	80	80	90	90	**12.5**
9	90	80	80	95	**2.9**
10	75	55	80	75	**19.2**
11	5	0	5	-	**100**
12	100	100	99	100	**-0.5**
13	100	100	100	100	**0**
14	100	100	60	100	**-20**
15	20	-	30	20	**25**
16	80	-	98	95	**20.6**
17	85	-	90	95	**8.8**
18	80	80	80	-	**0**
**Mean improvement (%)**					**9.8**
